# Handbook of Eclampsia

**Published:** 1884-07

**Authors:** 


HANDBOOK OF ECLAMPSIA OR NOTES AND CASES OF PUERPERAL CONVULSIONS. Comprising all the cases which have occurred during the present century, within a radius of several miles around Avondale, Chester Co., Penn., so far as can be ascertained, by E. Michener, M. D., J. H. Stubbs, M. D., B. Thompson, M. D, R. B. Ewing, M. D., S. Stebbins, M. D., Philadelphia, F. A. Davis, Attorney, 1217 Filbert street, 1883. 16 mo. pp. 68.

The object of this little book is to encourage blood-letting in Puerperal Eclampsia.

It is a history of forty-four cases occurring "within a radius of several miles around Avondale, as a centre, during the present century, a territory of nearly two hundred square miles and a period of more than four-score years."

It should receive consideration from obstetricians and general practitioners.

